# In Vitro Inhibition of Pathogens by Polyols: Optical Density-Based Screening and Implications for the Oral–Systemic Axis

**DOI:** 10.3390/microorganisms14040884

**Published:** 2026-04-15

**Authors:** Mark Cannon, Bradley S. Stevenson

**Affiliations:** 1 Ann and Robert H. Lurie Children’s Hospital of Chicago, Chicago, IL 60611, USA; 2 Department of Earth, Environmental and Planetary Sciences, Northwestern University, Evanston, IL 60208, USA; bradley.stevenson@northwestern.edu

**Keywords:** polyols, allulose, erythritol, xylitol, D-mannose, oral microbiome, *Streptococcus mutans*, *Streptococcus anginosus*, *Fusobacterium nucleatum*, *Candida albicans*, oral–systemic link

## Abstract

Polyols are widely used as non-cariogenic sweeteners in foods and oral care products, yet their comparative activity against diverse oral microbes and their potential relevance to the oral–systemic axis remain incompletely defined. Here, we performed an in vitro, optical density (OD)-based screening of four polyols—allulose, D-mannose, erythritol, and xylitol—against *Streptococcus mutans*, *Streptococcus anginosus*, *Candida albicans*, and *Fusobacterium nucleatum*. Cultures were grown with polyols at 1–20% (*w*/*v*), and OD600 was recorded at organism-specific endpoints (~24 h). Allulose, erythritol, and xylitol produced strong, concentration-dependent suppression of streptococcal growth at ≥5–10%, whereas *C. albicans* showed minimal changes across the tested range. *F. nucleatum* was highly sensitive to allulose, D-mannose, and xylitol at ≥5% (reducing OD to ≤13% of the untreated control), while low concentrations of D-mannose and erythritol increased OD beyond that of the control, suggesting species-specific utilization or stress responses. One-way ANOVA with Tukey’s HSD post hoc testing supported significant between-polyol differences for most concentrations in *Streptococcus* spp. and *F. nucleatum*. Collectively, these results identify polyol- and taxon-specific growth phenotypes that can inform the formulation of swallow-safe oral hygiene products and motivate follow-up work in polymicrobial biofilm models and clinical studies targeting oral inflammation and downstream systemic risk.

## 1. Introduction

Dental caries and periodontal diseases are highly prevalent, microbially mediated conditions that contribute substantially to pain, tooth loss, and healthcare costs. In the United States, surveillance data continue to show a high burden of untreated or treated caries across the lifespan [1], and population-based studies have documented a high prevalence of periodontitis in adults, including older adults [2,3].

Because dietary fermentable carbohydrates favor acidogenic and aciduric taxa and promote dysbiotic biofilms, non-fermentable sweeteners are attractive prevention tools. Polyols such as xylitol and erythritol have been incorporated into chewing gum, lozenges, toothpaste, and mouth rinse formulations. Clinical and mechanistic studies support their non-cariogenic nature and their caries-preventive effects [4,5,6].

Xylitol can inhibit the growth and acid production of mutans streptococci through intracellular accumulation of xylitol-5-phosphate and a futile phosphorylation–dephosphorylation cycle that disrupts central metabolism [7,8]. In mixed oral communities, replacing fermentable sugars with polyols can shift community metabolism and reduce acidification [9,10]. Erythritol has also been associated with anti-caries effects and can influence oral biofilm characteristics, with some studies suggesting distinct activity profiles relative to xylitol [11,12]. Combination approaches may further modulate microbial phenotypes and biofilm formation [13]. Beyond dental endpoints, polyols have been discussed in the context of broader host–microbe interactions and systemic health [14]. Polyols are naturally found in berries and other commercial foods, such as plums and strawberries [15].

Interest is expanding to additional low-calorie sweeteners and rare sugars, including allulose (D-psicose), which has gained attention for metabolic applications and shows emerging activity against selected oral taxa [16,17]. D-mannose, although not typically considered an oral health ingredient, is widely studied as a non-antibiotic approach to urinary tract infection prophylaxis and may influence microbial adhesion and carbohydrate utilization pathways in species-specific ways [18,19].

These compounds also reach the oral cavity through different edible or swallowable formats. Xylitol and erythritol are commonly delivered in chewing gums, mints, lozenges, saliva stimulants, and other sugar-free products, whereas allulose is an emerging rare sugar sweetener, and D-mannose is more often consumed as a supplement than as a traditional oral-health ingredient [4,5,6,11,12,13,14,15,16,17,18,19]. Concentrations of these polyols in oral healthcare products range from 3% to 25%, with at least 10% of a polyol, such as xylitol, considered the standard for efficacy [6]. This distinction matters for translation. The concentrations tested in vitro are defined as broth concentrations, whereas in vivo exposure depends on the grams per serving, salivary dilution, contact time, and frequency of use. Accordingly, lower assay concentrations are likely more representative of transient whole-mouth exposure, whereas the highest concentrations may better approximate short-lived product microenvironments. Recently, both allulose and D-mannose have been added to oral hygiene products.

Importantly, the oral microbiome and periodontal inflammation are increasingly viewed through an oral–systemic framework in which local dysbiosis can contribute to systemic inflammatory tone, transient bacteremia, and gut exposure to swallowed oral microbes and microbial products [20,21,22,23,24,25]. The organisms evaluated here have relevance beyond the oral cavity: *Streptococcus mutans* has been linked to cardiovascular complications such as infective endocarditis and, in certain strain backgrounds, cerebrovascular pathology [26,27]; the *Streptococcus anginosus* group can act as opportunistic pathogens in extraoral infections [28]; and *Fusobacterium nucleatum* is a key periodontal/peri-implant organism with documented invasive and immunomodulatory properties and has been discussed in the context of gastrointestinal disease processes [29,30,31].

The aim of this study was to conduct a comparative concentration–response screening of four polyols (allulose, D-mannose, erythritol, and xylitol) against representative oral bacteria and yeast using a standardized optical density assay. We hypothesized that growth inhibition would be polyol- and taxon-specific and would increase with concentration, providing a quantitative starting point for the rational design of swallow-safe oral hygiene formulations and for subsequent biofilm and translational studies.

## 2. Materials and Methods

### 2.1. Microorganisms and Culture Conditions

The following microorganisms were evaluated: *Streptococcus mutans* (ATCC 25175), *Streptococcus anginosus* (ATCC 700231), *Candida albicans* (ATCC 18804), and *Fusobacterium nucleatum* subsp. *nucleatum* (ATCC 23726). Single colonies on solid agar medium, streaked from glycerol stocks at −80 °C, were used to inoculate liquid cultures. *Streptococcus* spp. were cultured in brain heart infusion (BHI) broth (Oxoid) at 37 °C with shaking at 250 rpm, *C. albicans* in yeast peptone dextrose (YPD) broth (BD Difco™, Fisher Scientific, Pittsburgh, PA, USA) at 30 °C with shaking at 250 rpm, and *F. nucleatum* in tryptic soy agar broth (TSA; BD Difco™, Fisher Scientific) at 37 °C without shaking. *F. nucleatum* was cultured under anaerobic conditions either in a vinyl anoxic chamber (Coy Lab Products, Grass Lake, MI, USA) or in 18 × 150 mm anaerobic tubes with a butyl rubber stopper and anoxic headspace [32].

### 2.2. Polyols and Preparation

Allulose (D-psicose), D-mannose, erythritol, and xylitol were tested at final concentrations of 0%, 1%, 2%, 5%, 10%, and 20% (*w*/*v*) in each respective medium. Polyols were prepared as 20% (*w*/*v*) stock solutions in the corresponding growth medium, filter-sterilized (0.22 µm), and mixed with fresh, filter-sterilized medium immediately before inoculation. This concentration range was selected to span the low-to-high screening conditions used in prior oral sweetener studies [9,13,15,16]. The pH of BHI (7.6), TSA (7.4), and YPD (6.7) decreased when amended with 20% (*w*/*v*) allulose (0.6 ± 0.02), erythritol (0.1 ± 0.04), D-mannose (0.7 ± 0.06), or xylitol (0.09 ± 0.04). However, the optimal pH for growth for each organism tested was well within these ranges [33].

### 2.3. Optical Density Growth Assay

Growth was quantified using optical density (OD) [34]. Briefly, overnight stationary-phase cultures were used as the primary inoculum at a 1:100 (*v*/*v*) dilution in growth medium containing the indicated polyol concentration; thus, the assay used a 1% inoculum from the overnight culture rather than a McFarland-standardized suspension. The endpoint OD screening format was adapted from prior sweetener studies in oral microorganisms [9,13,15,16]. Cultures were vortexed to mix, distributed into triplicate culture tubes (3 mL in 13 × 100 mm disposable borosilicate glass tubes for BHI and YPD; 10 mL in 18 × 150 mm stoppered anaerobic tubes for TSA), and incubated under organism-appropriate conditions. At each time point, cultures were vortexed to fully suspend biomass, and OD was measured spectrophotometrically at 600 nm using a Spectronic 200E spectrophotometer (Thermo Scientific, Pittsburgh, PA, USA). Endpoint OD values were recorded at approximately 24 h for each organism (*S. mutans*, 2.50 at 24.75 h; *S. anginosus*, 2.00 at 23.75 h; *C. albicans*, 0.838 at 23.5 h; *F. nucleatum*, 1.073 at 25.5 h). Each condition was measured in triplicate cultures (*n* = 3) within a run.

### 2.4. Data Processing and Normalization

For each organism, OD values are reported as mean ± standard deviation (SD) across triplicate cultures. For *F. nucleatum*, OD values are additionally expressed as a percentage of the untreated control (% of control = mean ODcondition/mean ODcontrol × 100). Small OD values in negative controls of *F. nucleatum* were due to the thicker glass of the anaerobic tubes used for growth and OD determination, as well as small scratches from their reuse. Where polyol-treated values were constant across replicates (SD = 0), this is explicitly reported.

### 2.5. Statistical Analysis

To compare polyols across concentrations, one-way analysis of variance (ANOVA) was performed for each organism and concentration, excluding the 0% control. Tukey’s honestly significant difference (HSD) post hoc test was then used to identify pairwise differences among polyols. A two-tailed significance threshold of *p* < 0.05 was used.

## 3. Results

### 3.1. Streptococcus mutans

**Figure 1:**
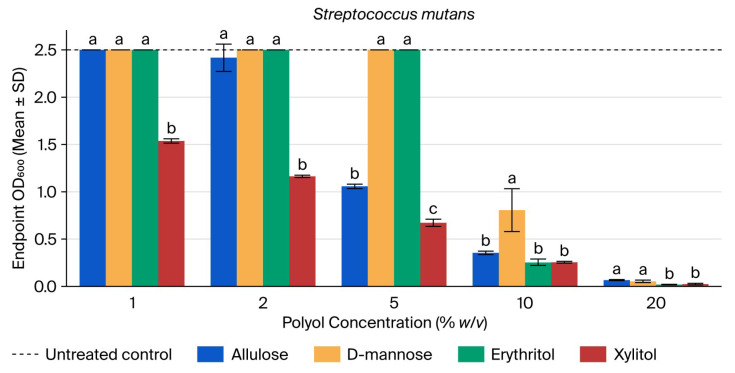
Endpoint OD600 of *Streptococcus mutans* at 24.75 h across polyol concentrations. Bars show mean ± SD (*n* = 3). Different letters within a concentration indicate Tukey’s HSD groupings (*p* < 0.05). The dashed line indicates the untreated control mean.

Untreated *S. mutans* cultures reached a mean endpoint OD600 of 2.50 at 24.75 h (see Figure 1). Allulose showed clear concentration-dependent suppression, beginning at 5% (OD600 = 1.057; 42% of control) and approaching near-complete inhibition at 20% (OD600 = 0.066; 3% of control). D-mannose and erythritol showed little reduction at 1–5% but strong inhibition at 10–20%. Xylitol reduced growth across the full concentration range, from OD600 = 1.537 (61% of control) at 1% to 0.024 (1% of control) at 20% (Supplementary Table S1).

Across polyols, one-way ANOVA was significant at every tested concentration (1%: F = 5259.18, *p* < 0.001; 2%: F = 247.20, *p* < 0.001; 5%: F = 5573.18, *p* < 0.001; 10%: F = 15.86, *p* = 0.001; 20%: F = 22.17, *p* < 0.001). Tukey’s HSD showed that xylitol differed from all other polyols at 1% and 2%; allulose occupied an intermediate position between the higher D-mannose/erythritol cluster and lower xylitol at 5%; D-mannose remained significantly higher than allulose, erythritol, and xylitol at 10%; and erythritol and xylitol were lower than allulose and D-mannose at 20% (Supplementary Table S2). The very large F value at 1% reflects a ceiling effect and near-zero within-group variance for allulose, D-mannose, and erythritol, whereas xylitol remained distinctly lower.

### 3.2. Streptococcus anginosus

**Figure 2:**
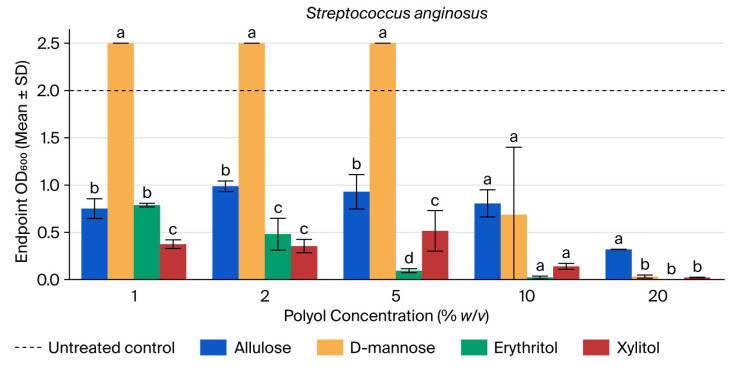
Endpoint OD600 of *Streptococcus anginosus* at 23.75 h across polyol concentrations. Bars show mean ± SD (*n* = 3). Different letters within a concentration indicate Tukey’s HSD groupings (*p* < 0.05). The dashed line indicates the untreated control mean.

At 23.75 h, the untreated control averaged an OD600 of 2.00 (see Figure 2). Allulose, erythritol, and xylitol reduced growth across the tested range, with erythritol showing near-complete inhibition at 10–20%. D-mannose displayed a biphasic pattern, exceeding the control at 1–5% and then falling sharply at 10–20% (Supplementary Table S3).

ANOVA across polyols was significant at 1% (F = 813.67, *p* < 0.001), 2% (F = 320.94, *p* < 0.001), 5% (F = 165.92, *p* < 0.001), and 20% (F = 276.99, *p* < 0.001), but not at 10% (F = 3.43, *p* = 0.073). Tukey’s HSD indicated that D-mannose remained significantly higher than the other polyols at 1–5%, whereas allulose was significantly higher than D-mannose, erythritol, and xylitol at 20%. No pairwise contrasts remained significant at 10% after correction (Supplementary Table S4).

### 3.3. Candida albicans

**Figure 3:**
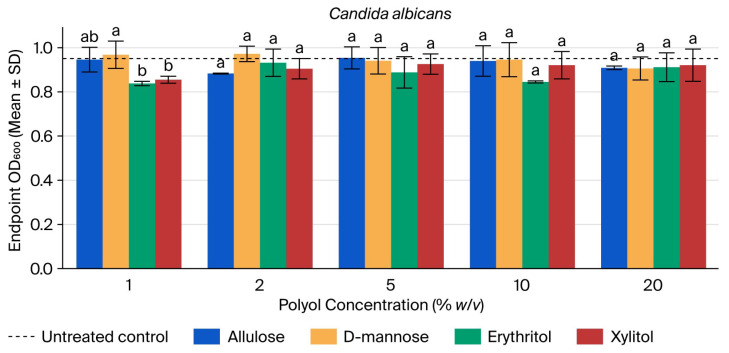
Endpoint OD600 of *Candida albicans* at 23.5 h across polyol concentrations. Bars show mean ± SD (*n* = 3). Different letters within a concentration indicate Tukey’s HSD groupings (*p* < 0.05). The dashed line indicates the untreated control mean.

*C. albicans* showed comparatively modest changes in endpoint OD600 at 23.5 h across the tested range (see Figure 3). Allulose and D-mannose remained close to the untreated control across 1–20%, and xylitol likewise produced only small shifts. Erythritol yielded the largest relative decrease at 1% (OD600 = 0.838; 88% of control), but no clear concentration–response trend emerged (Supplementary Table S5).

ANOVA was significant only at 1% (F = 6.87, *p* = 0.013) and was not significant at 2%, 5%, 10%, or 20% (all *p* ≥ 0.138). Tukey’s HSD at 1% identified significant differences only between D-mannose and erythritol and between D-mannose and xylitol. No pairwise contrasts were significant at the other concentrations (Supplementary Table S6).

### 3.4. Fusobacterium nucleatum

**Figure 4:**
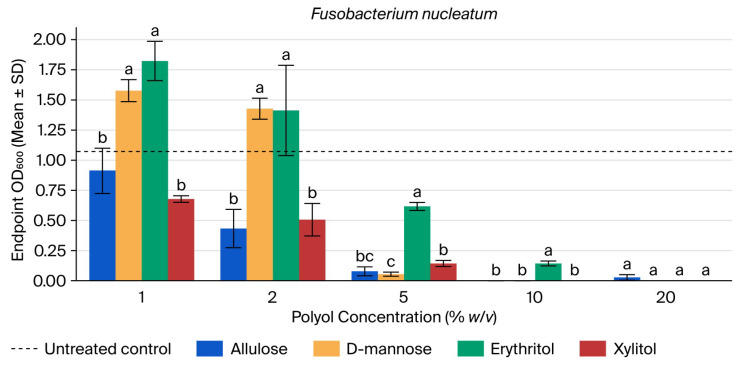
Endpoint OD600 of *Fusobacterium nucleatum* at 25.5 h across polyol concentrations. Bars show mean ± SD (*n* = 3). Different letters within a concentration indicate Tukey’s HSD groupings (*p* < 0.05). The dashed line indicates the untreated control mean. The untreated control mean OD was 1.070 +/− 0.485, whereas that of erythritol 1% was 1.823 +/− 0.163 and that of erythritol 2% was 1.413 +/− 0.374; for D-mannose 1%, the mean OD was 1.577 +/− 0.091, and for D-mannose 2%, it was 1.427 +/− 0.087.

*F. nucleatum* showed pronounced sensitivity to polyol exposure (see Figure 4). Relative to the untreated control (mean OD600 = 1.073), allulose, D-mannose, and xylitol reduced growth to ≤13% of control at 5% and to ~0% at 10%. Erythritol produced weaker suppression at 1–5% and remained the only condition with measurable residual growth at 10% (OD600 = 0.143; 13% of control; Supplementary Table S7).

ANOVA across polyols was significant at 1% (F = 49.33, *p* < 0.001), 2% (F = 18.96, *p* < 0.001), 5% (F = 250.56, *p* < 0.001), and 10% (F = 153.37, *p* < 0.001), with a marginal omnibus effect at 20% (F = 4.13, *p* = 0.048). Tukey’s HSD showed that allulose and xylitol were lower than D-mannose and erythritol at 1–2%; erythritol remained significantly higher than allulose, D-mannose, and xylitol at 5%; and erythritol was the only group differing at 10%, where allulose, D-mannose, and xylitol were uniformly at 0. No pairwise contrasts remained significant at 20% after correction (Supplementary Table S8).

## 4. Discussion

This study addresses a practical gap between the widespread use of polyol-containing edible/oral products and the limited availability of standardized, side-by-side screening data across multiple oral taxa [4,5,6,7,8,9,10,11,12,13,14,15,16,17,18]. We compared allulose, D-mannose, erythritol, and xylitol in a common OD-based assay and interpreted the results within an oral–systemic framework [20,21,22,23,24,25]. The discussion below focuses on how these organism-specific growth phenotypes align with the prior literature, whether they are formulation-relevant, and why they should still be considered as screening-level findings rather than clinical outcomes.

### 4.1. Summary of Main Findings

Using a standardized OD600 endpoint screen, we observed clear concentration-dependent growth suppression of *Streptococcus* spp. and *F. nucleatum*, whereas *C. albicans* changed little across the tested range.

Allulose showed the broadest inhibitory profile among the bacteria. Erythritol and xylitol were especially active against *S. anginosus* and *F. nucleatum*. D-mannose showed the strongest organism dependence, including apparent growth enhancement at low concentrations for *S. anginosus* and *F. nucleatum*. These patterns indicate that polyols are not functionally interchangeable and that response depends strongly on the target organism.

### 4.2. Relationship to Prior Literature on Xylitol and Erythritol

The inhibitory activity of xylitol against cariogenic streptococci has been documented for decades and is commonly linked to phosphotransferase-dependent uptake, intracellular accumulation of xylitol-5-phosphate, and a futile cycle that burdens central metabolism [7,8]. Consistent with that framework, clinical and experimental studies have reported reductions in mutans streptococci and cariogenic activity, although the magnitude of the reductions depends on dose, exposure frequency, and baseline risk [4,5,6]. In our dataset, xylitol strongly suppressed *S. anginosus* and *F. nucleatum* at higher concentrations.

Erythritol has likewise been associated with anti-caries effects and with changes in adherence and biofilm structure [11,12,13]. Our results extend that pattern by showing near-complete suppression of *S. anginosus* at 10–20% and strong inhibition of *F. nucleatum* at 10%, while *C. albicans* remained comparatively insensitive. Taken together, the data support overlap between xylitol and erythritol but also suggest distinct taxon-specific activity profiles.

### 4.3. Emerging Evidence for Allulose and D-Mannose in Oral Microbial Modulation

Allulose (D-psicose) is a rare sugar that has attracted increasing interest in metabolic and microbiome research. Recent work has begun to evaluate its impact on oral taxa, including effects on *F. nucleatum*-associated carbohydrate metabolism and on *S. mutans* acid production [15,16]. Our screening results are consistent with the premise that allulose can impose inhibitory pressure across multiple oral bacterial taxa, with particularly strong effects on *F. nucleatum* at ≥5%.

By contrast, D-mannose is more often discussed as a non-antibiotic strategy for recurrent urinary tract infections and as an adhesion-modulating carbohydrate rather than as a conventional oral health ingredient [17,18]. The biphasic patterns observed here, including apparent growth enhancement at low concentrations followed by inhibition at higher concentrations, suggest that D-mannose and other polyols may act as a utilizable substrate or signaling cue for some taxa at low exposure levels but may shift toward osmotic or metabolic stress at higher exposure levels. Distinguishing among these possibilities will require complementary assays, including CFU enumeration, pH and metabolite profiling, transcriptomics, and defined-medium growth studies.

### 4.4. Implications for Oral Biofilms and Formulation Design

Although planktonic OD assays provide a practical first-pass screen, oral microorganisms in vivo exist primarily in structured, polymicrobial biofilms embedded in an extracellular matrix and exposed to saliva, host factors, and dynamic pH gradients. Carbohydrate substitution can reshape mixed-community metabolism, and prior work has shown that polyols can alter the composition and metabolic output of mixed oral cultures relative to glucose [10]. Translation of these findings, therefore, requires consideration not only of growth inhibition but also of acidogenesis, extracellular polysaccharide production, adherence, and interspecies interactions.

Biofilms are clinically important because matrix-protected communities sustain cariogenic and inflammatory microenvironments, increase tolerance to environmental stress, and contribute to recurrent disease. In this context, biofilm-directed effects may influence caries progression, gingivitis, periodontitis, peri-implant disease, halitosis, and downstream treatment burden, including repeated restorative care, periodontal therapy, antimicrobial exposure, and management of patients at risk for bacteremia or chronic inflammation [20,21,22,23,24,25,29,30].

From a formulation standpoint, the in vitro concentrations tested here should be compared with caution to real-world exposure. Xylitol and erythritol are commonly consumed in gums, lozenges, mints, and saliva stimulants, whereas D-mannose is more often taken as a supplement, and allulose is an emerging sweetener in low-calorie foods [4,5,6,11,12,13,14,15,16,17,18]. Accordingly, the 10–20% (*w*/*v*) conditions likely represent upper-bound local microenvironments near dissolving tablets, gels, or lozenges, whereas 1–5% may better approximate transient whole-mouth exposures before salivary clearance. Future work should therefore incorporate short-exposure and repeated-exposure paradigms that better reflect in vivo kinetics.

### 4.5. Strengthening the Oral–Systemic Discussion: Mechanistic Pathways and Organism-Specific Relevance

The oral–systemic link is increasingly viewed as a set of interacting pathways through which oral dysbiosis and periodontal inflammation influence distal physiology. Current models emphasize the dissemination of inflammatory mediators from inflamed periodontal tissues, episodic bacteremia, and microbial product translocation during routine oral activities, as well as the oral–gut axis, in which swallowed oral microbes and inflammatory exudate perturb gut ecology, barrier function, and immune tone [19,20,21,22,23].

Experimental and translational studies further suggest that oral pathogens can disrupt epithelial junctions, interfere with immune function, promote thrombosis and atherogenesis, and contribute to pro-tumor or pro-senescent signaling in susceptible tissues [24]. These mechanisms help explain why oral dysbiosis is increasingly discussed as part of broader systemic risk rather than as an isolated dental problem.

Within this framework, the taxa evaluated here have clinically relevant features. Oral streptococci are common contributors to infective endocarditis, and subsets of *S. mutans* strains carrying collagen-binding adhesins such as Cnm have been associated with cerebrovascular pathology [26,27]. The *Streptococcus anginosus* group is notable for abscess formation and opportunistic infection after translocation to sterile sites [27]. *F. nucleatum* is a bridging organism in periodontal biofilms and can invade epithelial cells, modulate host immunity, and contribute to peri-implant disease; it has also been discussed in gastrointestinal disease and cancer-associated microbiomes [29,30,31,35].

From a translational standpoint, interventions that reduce the growth or cariogenic/periodontopathogenic activity of oral microbes could plausibly lower the frequency or magnitude of bacteremia and inflammatory signaling. However, the present study is an in vitro, single-species growth screen. It does not directly measure biofilm dysbiosis, periodontal inflammation, bacteremia, or systemic endpoints. Oral–systemic implications should therefore be interpreted as a mechanistic rationale rather than demonstrated outcomes. Logical next steps include polymicrobial biofilm models, host–microbe co-culture systems, and clinical studies that pair oral outcomes (e.g., plaque pH, gingival inflammation, salivary biomarkers) with systemic biomarkers in at-risk populations [20,21,22,23,24,25]. Selected examples linking oral microbes and systemic processes are summarized in Table 1.

### 4.6. Safety Considerations for Swallow-Safe Oral Products

Polyols have a long history of use in foods and dental products. Their safety and tolerability, however, depend on dose, route, and host factors. Gastrointestinal symptoms such as bloating or osmotic diarrhea can occur with higher oral intakes, although adaptation is often reported [40]. Absorption and metabolism also differ among compounds. Xylitol is more readily absorbed and metabolized than erythritol, whereas erythritol is largely absorbed and excreted unchanged in urine [41]. Reviews of metabolic effects remain mixed and are highly compound-specific [42].

Allulose has undergone regulatory review and has been evaluated in recent human intake studies, supporting continued interest in swallow-safe formulations that use rare sugars as functional excipients [43,44].

For xylitol specifically, clinical trials have investigated pediatric applications such as otitis media prevention, supporting the practical feasibility of defined swallowable dosing regimens [45,46]. At the same time, newer work has raised questions about possible prothrombotic associations with high circulating xylitol levels in selected settings [47]. The relevance of those findings to topical oral-care exposures—where a substantial fraction may be expectorated, and systemic exposure is far lower—is questionable.

### 4.7. Limitations

Several limitations should be noted. First, OD600 is a turbidity measure and can be influenced by cell size, aggregation, refractive index, and high-solute optical effects. Second, endpoint OD does not distinguish between bacteriostatic and bactericidal activity and does not capture biofilm formation, viability, or metabolic outputs, such as acid production.

Third, the dataset includes triplicate culture tubes per run, and the extent of independent biological replication should be clarified in the final report. Fourth, polyol concentrations and exposure durations in vivo differ substantially from those used in a 24 h in vitro incubation. Therefore, these data should be interpreted as screening-level results that motivate more physiologically representative models.

### 4.8. Future Directions

Future studies should (i) validate growth effects using CFU-based viability assays and/or flow cytometry; (ii) quantify acidogenesis and pH dynamics, particularly for cariogenic taxa; (iii) evaluate multi-species biofilms formed in saliva or saliva-mimicking media; (iv) test clinically relevant exposure paradigms (short, repeated exposures); and (v) assess impacts on commensal taxa to avoid unintended dysbiosis. Given the oral–systemic context, clinical studies in at-risk groups could incorporate systemic biomarkers (e.g., CRP, HbA1c, endothelial activation markers) alongside oral inflammation measures to evaluate whether microbiome-targeted polyol formulations confer benefits beyond local oral outcomes [20,21,22,23,24,25].

## 5. Conclusions

This in vitro OD-based screening suggests that polyols and rare sugars can exert strong, taxon-specific growth phenotypes against oral microbes. Allulose and erythritol displayed broad inhibitory activity against *Streptococcus* spp., while *F. nucleatum* was highly sensitive to allulose, D-mannose, and xylitol at ≥5%. By contrast, *C. albicans* changed little across the tested range. Because real-world exposures from foods, lozenges, gums, and related products are transient and formulation-dependent, the present concentrations should be interpreted as a screening framework rather than a direct in vivo proxy. These findings nevertheless provide a useful basis for biofilm-focused and clinically oriented studies of microbiome-targeted polyol formulations.

## Figures and Tables

**Table 1:** Selected examples of oral–systemic connections relevant to taxa evaluated in this study. Evidence strength varies across conditions.

Systemic Condition/Pathway	Example Oral Microbial Connection (Selected Examples)
Cardiometabolic inflammation and atherosclerotic risk	Periodontal inflammation can increase systemic inflammatory mediators and endotoxemia; episodic bacteremia and oral–gut axis are proposed routes [20,21,22,23,24,25].
Infective endocarditis	Oral streptococci are common etiologic agents; routine activities can seed transient bacteremia [27].
Cerebrovascular disease	Cnm+ *S. mutans* strains have been associated with cerebrovascular pathology, including cerebral microbleeds [26].
Diabetes and metabolic dysregulation	Bidirectional links between periodontal inflammation and glycemic control have been proposed; oral–gut axis and systemic cytokines are implicated [20,21,22,23,24,25].
Adverse pregnancy outcomes	Periodontitis and oral dysbiosis have been associated with adverse outcomes in observational and meta-analytic studies [36]
Neurodegeneration	Oral pathogens and periodontal inflammation have been investigated in relation to Alzheimer’s disease; microbial and inflammatory mechanisms are discussed in translational work [37,38]
Non-alcoholic fatty liver disease	Oral dysbiosis may influence the gut–liver axis through swallowed microbes and immune modulation [39].
Gastrointestinal disease and malignancy-associated microbiomes	*F. nucleatum* has been discussed in gastrointestinal disease contexts and cancer-associated microbiomes, motivating interest in oral reservoirs [29,30,31]
Opportunistic invasive infections	The *Streptococcus anginosus* group can participate in deep-seated abscess formation following translocation [27].
Peri-implant and periodontal tissue destruction	*F. nucleatum* contributes to periodontal/peri-implant biofilms and can modulate host immune responses [29,30].

Examples are based on narrative and review literature on the oral microbiome, periodontal inflammation, and systemic disease pathways [20,21,22,23,24,25,26,27,28,29,30,31,32,33,34,35,38,37,36,39].

## Data Availability

The summary data and post hoc analysis supporting the findings of this study are provided in Supplementary File S1. Additional raw OD600 readings are available from the corresponding author upon reasonable request.

## Supplementary Materials

The following supporting information can be downloaded at: https://www.mdpi.com/article/10.3390/microorganisms14040884/s1, Supplementary File S1: Tables S1–S8: Endpoint OD summary tables and ANOVA/Tukey’s HSD post hoc results for all organisms.

## Abbreviations

ANOVA, analysis of variance; BHI, brain heart infusion; CRP, C-reactive protein; IL, interleukin; OD, optical density; OD600, optical density measured at 600 nm; PTS, phosphoenolpyruvate-dependent phosphotransferase system; SD, standard deviation; TSA, tryptic soy agar; TNF, tumor necrosis factor; YPD, yeast peptone dextrose.
