# Supplementary material for: In Vitro Inhibition of Pathogens by Polyols: Optical Density-Based Screening and Implications for the Oral–Systemic Axis
Source: Microorganisms. 2026 Apr 15;14(4):884. doi: 10.3390/microorganisms14040884 (PMC13118738; doi:10.3390/microorganisms14040884)
Supplement: Supplementary file 1 [file microorganisms-14-00884-s001.zip › microorganisms-4208168-supplementary.pdf]

Supplementary File S1. Endpoint OD summary tables and ANOVA/Tukey HSD post hoc results

This supplementary file contains the endpoint OD600 summary tables from the main manuscript, together with one-way ANOVA and Tukey's HSD post hoc summaries used for the lettering in Figures 1–4.

Table S1. *Streptococcus mutans* endpoint OD600 at 24.75 h (mean ± SD; n = 3). Values in parentheses are % of the untreated control (0% polyol).

Treatment	0%	1%	2%	5%	10%	20%
Control (0% polyol)	2.500 ± 0.000 (100%)	—	—	—	—	—
Allulose	—	2.500 ± 0.000 (100%)	2.417 ± 0.144 (97%)	1.057 ± 0.023 (42%)	0.354 ± 0.018 (14%)	0.066 ± 0.006 (3%)
D-mannose	—	2.500 ± 0.000 (100%)	2.500 ± 0.000 (100%)	2.500 ± 0.000 (100%)	0.806 ± 0.226 (32%)	0.053 ± 0.013 (2%)
Erythritol	—	2.500 ± 0.000 (100%)	2.500 ± 0.000 (100%)	2.500 ± 0.000 (100%)	0.252 ± 0.034 (10%)	0.019 ± 0.003 (1%)
Xylitol	—	1.537±0.023 (61%)	1.163±0.012 (47%)	0.672±0.038 (27%)	0.255±0.009 (10%)	0.024 ± 0.008 (1%)

Abbreviations: OD, optical density. Percent of control was calculated using the untreated control mean OD600 = 2.50.

Table S2. One-way ANOVA and Tukey's HSD post hoc comparisons for *Streptococcus mutans* (0% control excluded).

Concentration	ANOVA F	ANOVA p	Tukey HSD significant contrasts / summary
1%	5259.18	1.623e-13	Allulose vs Xylitol (p = 0.000e+00); D-mannose vs Xylitol (p = 0.000e+00); Erythritol vs Xylitol (p = 0.000e+00)
2%	247.20	3.179e-08	Allulose vs Xylitol (p = 0.000e+00); D-mannose vs Xylitol (p = 0.000e+00); Erythritol vs Xylitol (p = 0.000e+00)
5%	5573.18	1.288e-13	Allulose vs D-mannose (p = 0.000e+00); Allulose vs Erythritol (p = 0.000e+00); Allulose vs Xylitol (p = 0.000e+00); D-mannose vs Xylitol (p = 0.000e+00); Erythritol vs Xylitol (p = 0.000e+00)
10%	15.86	0.001	Allulose vs D-mannose (p = 0.0057); D-mannose vs Erythritol (p = 0.0016); D-mannose vs Xylitol (p = 0.0017)
20%	22.17	0.0003	Allulose vs Erythritol (p = 0.0006); Allulose vs Xylitol (p = 0.0012); D-mannose vs Erythritol (p = 0.0047); D-mannose vs Xylitol (p = 0.0118)

Group lettering in the main-text figures was derived from Tukey's HSD comparisons within each concentration. High F values in ANOVA indicate significant differences between group means, suggesting that the variation among the group means is greater than the variation within the groups.

Table S3. ***Streptococcus anginosus*** **endpoint OD600 at 23.75 h (mean ± SD; n = 3). Values in parentheses are % of the untreated control (0% polyol).**

Treatment	0%	1%	2%	5%	10%	20%
Control (0% polyol)	2.000 (100%)	—	—	—	—	—
Allulose	—	0.752 ± 0.104 (38%)	0.988 ± 0.056 (49%)	0.930 ± 0.182 (46%)	0.805 ± 0.144 (40%)	0.319 ± 0.002 (16%)
D-mannose	—	2.500 ± 0.000 (125%)	2.500 ± 0.000 (125%)	2.500 ± 0.000 (125%)	0.687 ± 0.714 (34%)	0.032 ± 0.016 (2%)
Erythritol	—	0.788 ± 0.019 (39%)	0.481 ± 0.168 (24%)	0.094 ± 0.021 (5%)	0.023 ± 0.014 (1%)	0.000 ± 0.000 (0%)
Xylitol	—	0.375 ± 0.046 (19%)	0.355 ± 0.070 (18%)	0.516 ± 0.215 (26%)	0.140 ± 0.032 (7%)	0.022 ± 0.027 (1%)

Percent of control was calculated using the untreated control mean OD600 = 2.00.

Table S4. One-way ANOVA and Tukey's HSD post hoc comparisons for *Streptococcus anginosus* (0% control excluded).

Concentration	ANOVA F	ANOVA p	Tukey HSD significant contrasts / summary
1%	813.67	2.799e-10	Allulose vs D-mannose (p = 0.000e+00); Allulose vs Xylitol (p = 0.0002); D-mannose vs Erythritol (p = 0.000e+00); D-mannose vs Xylitol (p = 0.000e+00); Erythritol vs Xylitol (p = 0.0001)
2%	320.94	1.131e-08	Allulose vs D-mannose (p = 0.000e+00); Allulose vs Erythritol (p = 0.0008); Allulose vs Xylitol (p = 0.0002); D-mannose vs Erythritol (p = 0.000e+00); D-mannose vs Xylitol (p = 0.000e+00)
5%	165.92	1.531e-07	Allulose vs D-mannose (p = 0.000e+00); Allulose vs Erythritol (p = 0.0004); Allulose vs Xylitol (p = 0.0291); D-mannose vs Erythritol (p = 0.000e+00); D-mannose vs Xylitol (p = 0.000e+00); Erythritol vs Xylitol (p = 0.0265)
10%	3.43	0.0727	No Tukey-adjusted pairwise differences.
20%	276.99	2.027e-08	Allulose vs D-mannose (p = 0.000e+00); Allulose vs Erythritol (p = 0.000e+00); Allulose vs Xylitol (p = 0.000e+00)

Group lettering in the main-text figures was derived from Tukey's HSD comparisons within each concentration.

Table S5. ***Candida albicans*** **endpoint OD600 at 23.5 h (mean ± SD; n = 3). Values in parentheses are % of the untreated control (0% polyol). The reported values are after 1/10 dilutions to generate ODs within the instrument's working range; the relative values remain the same.**

Treatment	0%	1%	2%	5%	10%	20%
Control (0% polyol)	0.951 ± 0.077 (100%)	—	—	—	—	—
Allulose	—	0.946 ± 0.056 (99%)	0.883 ± 0.002 (93%)	0.954 ± 0.050 (100%)	0.940 ± 0.069 (99%)	0.909 ± 0.008 (96%)
D-mannose	—	0.968 ± 0.062 (102%)	0.972 ± 0.035 (102%)	0.941 ± 0.060 (99%)	0.946 ± 0.077 (99%)	0.906 ± 0.052 (95%)
Erythritol	—	0.838 ± 0.010 (88%)	0.932 ± 0.062 (98%)	0.888 ± 0.071 (93%)	0.845 ± 0.005 (89%)	0.912 ± 0.065 (96%)
Xylitol	—	0.855±0.016 (90%)	0.905±0.046 (95%)	0.926 ± 0.061 (97%)	0.921±0.062 (97%)	0.921 ± 0.073 (97%)

Percent of control was calculated using the untreated control mean OD600 = 0.951.

Table S6. One-way ANOVA and Tukey's HSD post hoc comparisons for ***Candida albicans*** **(0% control excluded).**

Concentration	ANOVA F	ANOVA p	Tukey HSD significant contrasts / summary
1%	6.87	0.0133	D-mannose vs Erythritol (p = 0.0244); D-mannose vs Xylitol (p = 0.048)
2%	2.45	0.1381	No Tukey-adjusted pairwise differences.
5%	0.66	0.6001	No Tukey-adjusted pairwise differences.
10%	1.79	0.2273	No Tukey-adjusted pairwise differences.
20%	0.04	0.9881	No Tukey-adjusted pairwise differences.

Group lettering in the main-text figures was derived from Tukey's HSD comparisons within each concentration.

Table S7. ***Fusobacterium nucleatum*** **endpoint OD600 at 25.5 h (mean ± SD; n = 3). Values in parentheses are % of the untreated control (0% polyol).**

Treatment	1%	2%	5%	10%	20%
Allulose	0.915 ± 0.188 (85%)	0.433 ± 0.159 (40%)	0.078 ± 0.037 (7%)	0.000 ± 0.000 (0%)	0.027 ± 0.023 (3%)
D-mannose	1.577 ± 0.091 (147%)	1.427 ± 0.087 (133%)	0.054 ± 0.017 (5%)	0.000 ± 0.000 (0%)	0.000 ± 0.000 (0%)
Erythritol	1.823 ± 0.163 (170%)	1.413 ± 0.374 (132%)	0.617 ± 0.033 (58%)	0.143 ± 0.020 (13%)	0.000 ± 0.000 (0%)
Xylitol	0.678 ± 0.027 (63%)	0.507 ± 0.135 (47%)	0.143 ± 0.025 (13%)	0.000 ± 0.000 (0%)	0.000 ± 0.000 (0%)

Percent of control was calculated using the untreated control mean OD600 = 1.073.

Table S8. One-way ANOVA and Tukey's HSD post hoc comparisons for *Fusobacterium nucleatum* (0% control excluded).

Concentration	ANOVA F	ANOVA p	Tukey HSD significant contrasts / summary
1%	49.33	1.667e-05	Allulose vs D-mannose (p = 0.0013); Allulose vs Erythritol (p = 0.0001); D-mannose vs Xylitol (p = 0.0002); Erythritol vs Xylitol (p = 0.000e+00)
2%	18.96	0.0005	Allulose vs D-mannose (p = 0.0024); Allulose vs Erythritol (p = 0.0026); D-mannose vs Xylitol (p = 0.0038); Erythritol vs Xylitol (p = 0.0042)
5%	250.56	3.014e-08	Allulose vs Erythritol (p = 0.000e+00); D-mannose vs Erythritol (p = 0.000e+00); D-mannose vs Xylitol (p = 0.0232); Erythritol vs Xylitol (p = 0.000e+00)
10%	153.37	2.085e-07	Allulose vs Erythritol (p = 0.000e+00); D-mannose vs Erythritol (p = 0.000e+00); Erythritol vs Xylitol (p = 0.000e+00)
20%	4.13	0.0481	Omnibus ANOVA p = 0.048, but no Tukey-adjusted pairwise differences were retained.

Group lettering in the main-text figures was derived from Tukey's HSD comparisons within each concentration.
